# HFR Projector Camera Based Visible Light Communication System for Real-Time Video Streaming

**DOI:** 10.3390/s20185368

**Published:** 2020-09-19

**Authors:** Atul Sharma, Sushil Raut, Kohei Shimasaki, Taku Senoo, Idaku Ishii

**Affiliations:** 1 Department of System Cybernetics, Graduate School of Engineering, Hiroshima University, Hiroshima 739-0046, Japan; atul@robotics.hiroshima-u.ac.jp; 2 Digital Manufacturing Education Research Center, Hiroshima University, Hiroshima 739-0046, Japan; sushil@hiroshima-u.ac.jp (S.R.); simasaki@robotics.hiroshima-u.ac.jp (K.S.); 3 Graduate School of Advanced Science and Engineering, Hiroshima University, Hiroshima 739-0046, Japan; taku-senoo@hiroshima-u.ac.jp

**Keywords:** high speed vision, real-time video processing, visible light communication, wireless video streaming

## Abstract

This study develops a projector–camera-based visible light communication (VLC) system for real-time broadband video streaming, in which a high frame rate (HFR) projector can encode and project a color input video sequence into binary image patterns modulated at thousands of frames per second and an HFR vision system can capture and decode these binary patterns into the input color video sequence with real-time video processing. For maximum utilization of the high-throughput transmission ability of the HFR projector, we introduce a projector–camera VLC protocol, wherein a multi-level color video sequence is binary-modulated with a gray code for encoding and decoding instead of pure-code-based binary modulation. Gray code encoding is introduced to address the ambiguity with mismatched pixel alignments along the gradients between the projector and vision system. Our proposed VLC system consists of an HFR projector, which can project 590 × 1060 binary images at 1041 fps via HDMI streaming and a monochrome HFR camera system, which can capture and process 12-bit 512 × 512 images in real time at 3125 fps; it can simultaneously decode and reconstruct 24-bit RGB video sequences at 31 fps, including an error correction process. The effectiveness of the proposed VLC system was verified via several experiments by streaming offline and live video sequences.

## 1. Introduction

With the recent rapid advances in computer and image sensor technologies, many high frame rate (HFR) vision systems that can capture and process images simultaneously at thousands of frames per second have been developed [1,2,3,4,5]; many tracking algorithms, such as optical flow estimation [6,7], cam-shift tracking [8], and feature-point tracking [9], have been accelerated by the parallel implementation of these algorithms on field programmable gate array (FPGA) and graphics processing units. These HFR vision systems can have a large bandwidth that can simultaneously recognize high-speed phenomena, which are too fast to be recognized by the naked human eyes and standard video cameras operating at dozens of frames per second. Many vision-based dynamic sensing systems have been developed for the human-invisible dynamics of objects such as drone tracking [10,11], motion-blur-free video shooting [12,13,14,15], vibration analysis [16,17], and microscopic sensing [18,19,20,21]. In addition to these HFR vision systems that can capture the dynamic phenomena vibrating at hundreds or thousands of hertz, HFR projector systems based on the digital micro-mirror device (DMD) technology [22,23] can project binary image patterns at thousands of frames per second or more; several types of HFR projector–camera systems [24,25,26] have been reported in various applications, such as structured light based 3D sensing [27,28,29,30] and simultaneous projection mapping [31,32,33,34]. If a real-time HFR vision system could function as a communication receiver to perfectly capture and encode the HFR-blinking high-space-resolution image patterns with real-time video processing at thousands of frames per second, which are too fast for human eyes to see, and high-thorough visible light communication (VLC) could be realized for broadband video streaming by utilizing the high-resolution in mega-pixel order and high-frequency band in the kHz order in the HFR projection of an HFR projector.

In this study, we develop an HFR-projector–camera-based VLC system for broadband video streaming, in which a projector and vision system function as an image transmitter and image receiver operating at thousands of frames per second, respectively. We implemented video encoding and decoding processes based on a camera-projector-based VLC protocol for real-time video streaming so that the pixel-wise high-throughput light patterns projected from the HFR projector can be maximally utilized without losing any information when the spatial resolution of the HFR vision system is similar to that of the projector. The remainder of this paper is organized as follows. Section 2 summarizes the related works of VLC along with the problems in the conventional vision-based VLC system for broadband video streaming. Section 3 proposes the concept of the HFR-projector–camera-based VLC system. Section 4 and Section 5 provide an outline of the transmitter and receiver systems of the HFR-projector–camera-based VLC system, describing its VLC protocol that involves the video encoding and decoding processes based on gray code-based binary modulated light patterns which are robust to changing external illumination and unmatched pixel alignment between the vision system and projector, respectively. Section 6 describes the image quality parameter, and Section 7 presents the performance of real-time experiments conducted on several video sequences and live-camera video streaming, in which an HFR vision system can capture and process 512 × 512 images in real time at 3125 fps for 590 × 1060 binary images, which are modulated and projected from an HFR projector at 1041 fps. This system can simultaneously transmit and receive a 24-bit RGB 590 × 1060 video sequence at maximum 31 fps.

## 2. Related Works

VLC has emerged as an alternative technique to accommodate the exponentially increasing demands of radio frequency-based wireless communication [35,36,37,38]. The visible light corresponds to a band of frequencies ranging between 400 THz (750 nm) and 800 THz (375 nm) and is used as a source in VLC systems for transmitting encoded information using air as a transporting medium and decoded using an appropriate photoreceiver. The intensity of the light source is modulated according to the input data at a high rate which is faster than the persistence of human vision. However, a sensitive photodiode or an image sensor is used to detect the embedded information by decoding the on–off behavior of the light emitting diode (LED) [39,40,41,42,43,44]. In a VLC system, the image sensor has an advantage over the photodiode; it can separate information spatially and temporally from the light source, whereas the photodiode-based systems are highly sensitive to light and inexpensive, but require additional equipment for setting up a system. With the ability of image sensors to capture light, a new type of optical wireless communication is introduced known as camera communication, where image sensors are used for sensing the light intensity emitted from a light source [45,46]. Many potential applications of camera-based VLC systems include automotive systems [47,48,49], mobile phone-camera communications [50,51,52], indoor wireless communications [53,54,55], LED camera-based VLC [56,57], and image recognition and light signaling [58].

Image-sensors-based VLC systems have been developed to decode the information transmitted from different light sources, such as LEDs, display screens, and projectors. Various studies contributing to LED-camera based communication systems have been conducted, focusing on the rate of data transfer from the LED-to-camera and LED-based position location detection systems [59,60,61,62,63]. In addition, traffic signal LEDs are used for estimating the position of a vehicle using an in-vehicle camera and an LED-based VLC system [64,65,66,67]. The accuracy of LED-based systems depends on the number of LEDs used, focal length of the lens, pixel size, and frame rate of the camera receiver. To avoid the complexity of building an LED source circuit for transmission, a display screen or projector has been used as an alternative solution to increase the bit-rate transfer and overall speed of indoor-based VLC systems [68,69,70,71]. Display monitors and LCD panels modulate the screen intensities using different encoding techniques and are accordingly decoded using a camera at the receiver [72,73,74,75]. The data communication between a screen and a camera does not necessarily depend on the content of the screen, and it can be completely hidden from the user by integrating the information and content onto the screen, which further limits their application scenarios. The display screens and projectors with low frame rates make the communication systems slower and limited; this issue can be resolved by using HFR projectors that provide a high data transmission rate, in contrast to commercial projectors that support low frame rate projection and lack the controlling parameters.

The major drawback of conventional cameras and the cameras integrated in smartphones and tablets is that they all operate at a low frame rate due to which the communication bandwidth of VLC systems becomes low, which can be overcome by using an HFR camera. Therefore, we propose a VLC system with an HFR projector and HFR camera that can provide a higher communication bandwidth and better performance, as well as minimize the loss of information. This research mainly focuses on the spatio-temporal information which is similar to transmitting spatial information such as quick response codes (QR codes) and bar-codes; however, for transmitting the temporal information, LED-to-camera communication is considered, which decodes the data temporally. The processing of data in real time involves challenges, and, hence, additional information is embedded with transmitted video sequences for proper decoding at the receiver side. Thus, the spatio-temporal information is transmitted using an HFR projector which is then decoded spatially and temporally by an HFR camera that can be used in our system for transmitting real-time videos using VLC.

## 3. HFR Projector-Camera-Based VLC System

### 3.1. VLC System

**Figure 1:**
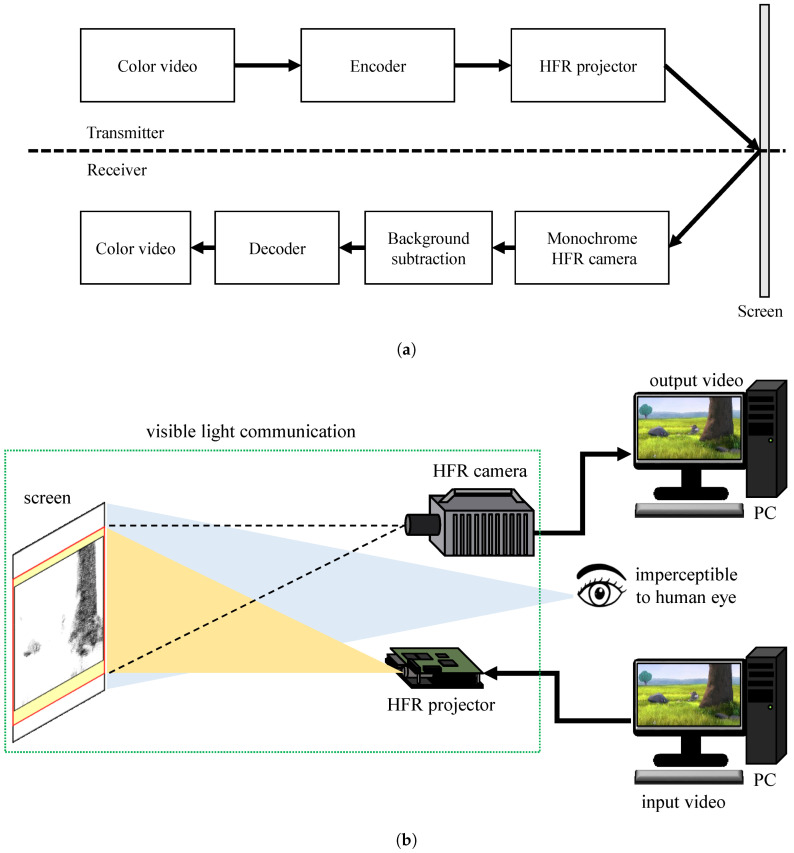
(**a**) block diagram and (**b**) configuration of the proposed VLC system.

This study introduces an HFR-projector–camera-based system for streaming videos in real time using VLC. There is some research based on projector–camera, but the projected content is perceptible to human eye, whereas the hidden encoded data is imperceptible. The drawback of these system is that they work at a low frame rate due to which the communication system becomes slower. The advantage of our system is that the data rate becomes higher by using the HFR projector and HFR camera. The system also explains the advantage of using gray-code coding over pure binary based coding and robustness to the ambient light. An overall block diagram of the proposed VLC system is shown in Figure 1, where an HFR projector can project the encoded stored color video sequences or universal serial bus (USB) camera videos into binary-modulated images that can be decoded using a monochrome HFR camera. The binary-modulated images are effectively decoded using additional information appended to each binary image as a header block that contains the current image information such as the frame number, starting of a new image, and channel bit plane information. In addition, the system can eliminate any ambiguities associated with mismatched pixel alignment along the gradient between the HFR projector and HFR camera using gray-code encoding instead of pure-binary-code-based image projection. At the receiver, the frame rate of the monochrome HFR camera is set to be thrice that of the HFR projector considering the Nyquist sampling rate so that the original projected image can be retrieved without any loss. The monochrome HFR camera captures the binary images to reconstruct the original image and background subtraction is performed for every captured binary image to make the system more robust against different textured backgrounds. In addition, the content of the cumulatively projected HFR binary images is imperceptible to human eyes, which results in secure data transmission.

### 3.2. System Configuration

An HFR projector is used as a transmitter to establish a high-speed VLC communication system with a high projection rate and control. The digital light processing (DLP) LightCrafter 4500 HFR projector uses a transmitter that provides a high projection rate of up to 4000 fps with bit plane projection control. DLP LightCrafter 4500 is a projection system with a two-dimensional array of electrically addressable and mechanically tiltable micro-mirrors to represent each pixel, known as digital mirror devices (DMDs), that are widely used in consumer electronics [76,77,78]. The DLP projector does not modulate the emitted wavelength of the projected light to reproduce the color intensity; instead, it reproduces by modulating the exposure time of the mirrors over a specific operating refresh time based on the projected frame bit planes. This projector supports 1-bit to 8-bit images with a resolution of 912 × 1140, and each pixel corresponds to a micro-mirror on the DMD. This feature helps with projecting data at the pixel level and transforms the image to be used for pixel-wise binary projection for the VLC system.

Dynamic changes related to HFR projection are imperceptible to human eyes and conventional cameras are unable to detect high-speed data or events. Therefore, to monitor high-speed phenomena continuously, we need HFR cameras to improve the shooting speed and performance. In this study, the proposed system consists of a monochrome HFR camera system that is an extension of Fastcam SA-X2 developed by Co. Photron and Hiroshima University; it provides a complementary metal oxide semiconductor (CMOS) sensor-based super high-speed vision platform that enables real-time image processing more than 10,000 fps against a megapixel image and global electronic shutter with excellent light sensitivity [4]. This camera is used as a receiver with an embedded external board that has an onboard FPGA for image processing; it produces output images with a resolution of 512 × 512 with a 12-bit dynamic range at 3125 fps in real time. This HFR camera system provides a high frame rate image capturing to meet the requirements of the proposed VLC system.

## 4. Transmitter Encoding System

**Figure 2:**
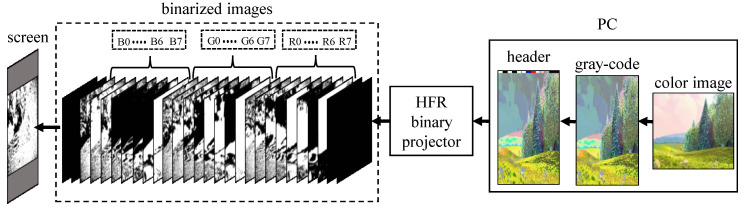
Transmitter.

The transmitter encoding system in the proposed VLC system has three stages: encoding image from pure-binary-code into gray-code, addition of header information, and binary image or bit-plane projection, as shown in Figure 2. The input RGB video is initially encoded frame-by-frame into gray-code from a pure-binary-code, and additional information, such as the frame number, along with other necessary information, is appended to the current image in the form of header information, which is then fed to the HFR projector, where it is deconstructed into binary images for projection.

### 4.1. Header Information

**Figure 3:**
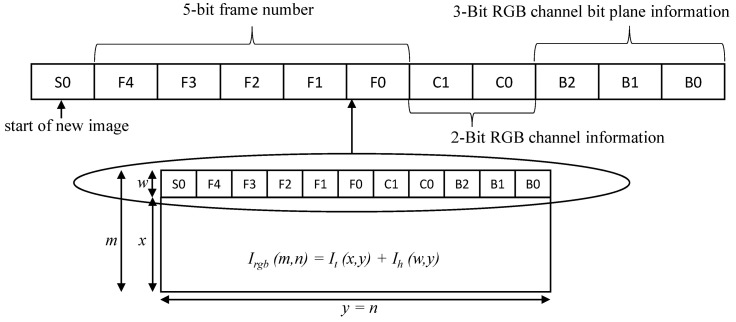
Header information.

The communication link between the transmitter and receiver is established by appending additional information of the image in the form of blocks of pixels as header information to the transmitting image. In the header, four blocks of pixels represent information about the current image, as shown in Figure 3, where the first block *S0*, whose all pixel values are set to a maximum value of 255 for an 8-bit pixel, is used for determining the start of a new image and software-based synchronization. The next five blocks of pixels (that is, *F4, F3, F2, F1,* and *F0*) in the header represent a 5-bit frame number ranging from 0 to 31, which is assigned to each frame continuously. Thereafter, 2-bit channel information is added using the *C1* and *C0* blocks to represent the red, green, and blue channels of an image, whereas the last 3-bits (that is *B2, B1,* and *B0*) represent eight bit-planes of a single channel that will be used to determine any loss in the bit-plane in an RGB channel of the image. The last five blocks of pixels help in determining the sequence of binary images for reconstructing an image. Therefore, let $I_{t}\left(x,y\right)$ be the input image, which is combined with the header information of size $I_{h}\left(w,y\right)$ to form a combined image, $I_{rgb}\left(m,n\right)$, before passing it to the HFR projector for binary image projection, as expressed in Equation (1):

(1) $$\;I_{rgb}\left(m,n\right)=I_{t}\left(x,y\right)+I_{h}\left(w,y\right)$$

### 4.2. Projection Pattern

**Figure 4:**
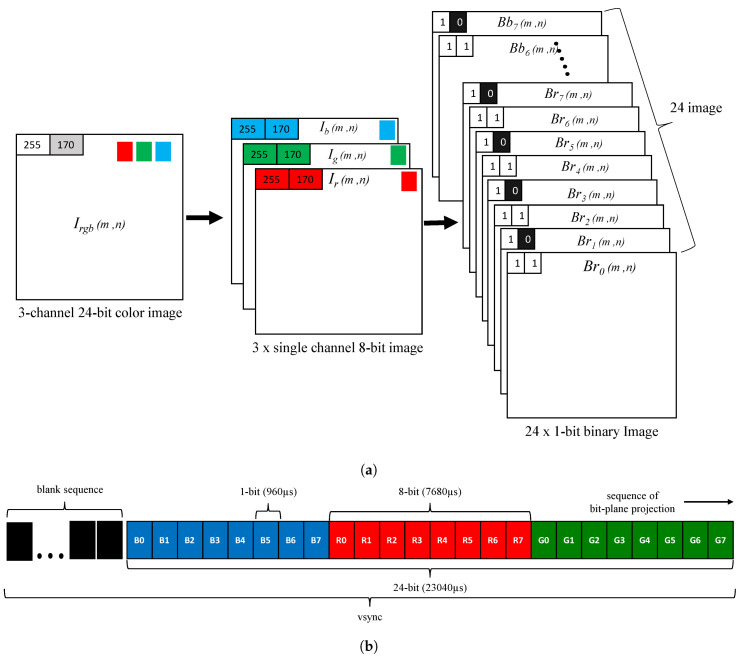
(**a**) decomposition of an RGB image into binary bit-plane images and (**b**) bit-plane projection pattern for a single RGB image.

The spatio-temporal projection of binary images by an HFR projector is achieved by decomposing a given packed 24-bit RGB image into its equivalent twenty-four 1-bit binary images. The HFR projector supports $2^{8}$= 256 intensity levels for an 8-bit channel and the decomposition of a 24-bit RGB color image is demonstrated in Figure 4a, where $I_{rgb}\left(m,n\right)$ is a three-channel 24-bit color image that is split into three single-channel 8-bit images, $I_{r}\left(m,n\right)$, $I_{g}\left(m,n\right)$ and $I_{b}\left(m,n\right)$. The 8-bit single-channel image is converted into binary images by the HFR projector as eight 1-bit images, where $Br_{t}\left(m,n\right)$, $Bg_{t}\left(m,n\right)$ and $Bb_{t}\left(m,n\right)$ represents the $t^{th}$ 1-bit image of the red, green, and blue channels, respectively; “t” represents the bit-plane number ranging between 0 and 7 for an 8-bit image. The projection sequence of binary images is defined by the users in the HFR projector controlling software, and the projection of a new image is triggered by vertical synchronization (vsync) signals. The pattern sequence for binary image projection is shown in Figure 4b, where the total duration of exposure for all patterns should be less than or equal to the vsync duration. The HFR projector introduces a sequence of blank images when the duration of all projection patterns is not equal to vsync.

### 4.3. Gray-Code Encoding

**Figure 5:**
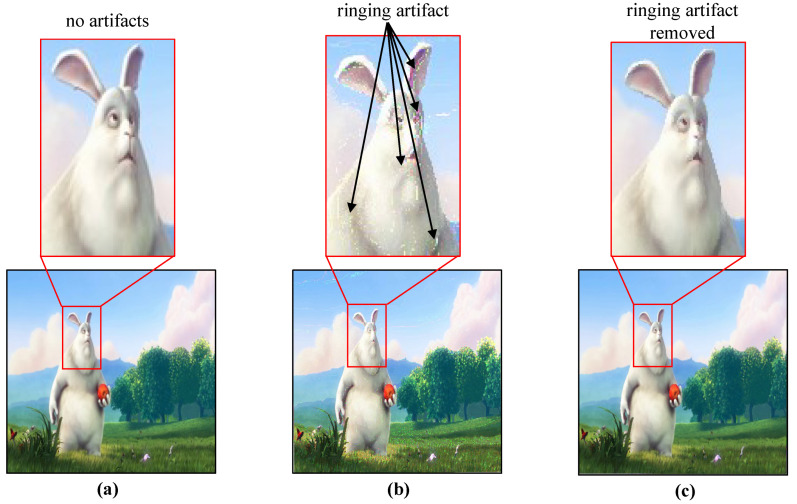
(**a**) original image; (**b**) reconstructed image with pure-binary-code; (**c**) reconstructed image with gray-code.

The image reconstructed with pure-binary-code has ambiguities along the gradients due to mismatched pixel alignment between the HFR projector and HFR camera which is overcome by gray-code-based projection. The ambiguities observed in the images reconstructed using pure-binary-code includes ringing artifacts as shown in Figure 5, which are reduced by gray-code-based image projection. Let $I_{t}\left(x,y\right)$ be the input RGB color image having three 8-bit channels as red $I_{r}\left(x,y\right)$, green $I_{g}\left(x,y\right)$, and blue $I_{b}\left(x,y\right)$ channel as expressed in Equation (2):

(2) $$\;I_{t}\left(x,y\right)=\left[\begin{array}{c} I_{r}\left(x,y\right)\\ I_{g}\left(x,y\right)\\ I_{b}\left(x,y\right) \end{array}\right]\;,$$

The pixel-value *P* of an input image is represented by a sequence of binary values ($b_{n-1}$, …, $b_{1}$, $b_{0}$) based on Equation (3). In an 8-bit image, each pixel is represented as eight 1-bit binary images where the higher bit-planes contain more significant visual information and the lower bit-plane shows more details. Using Equation (4), the gray-code representation of a binary pixel value *P*, is ($g_{n-1}$, …, $g_{1}$, $g_{0}$), which is used to convert the pure-binary-code images of red $I_{r}\left(x,y\right)$, green $I_{g}\left(x,y\right)$, and blue $I_{b}\left(x,y\right)$ channels into gray-code as $I_{gray_{r}}\left(x,y\right)$, $I_{gray_{g}}\left(x,y\right)$, and $I_{gray_{b}}\left(x,y\right)$, respectively, which are combined to make one 24-bit gray-code color image, $I_{gray_{t}}\left(x,y\right)$, as shown in Equation (5). The gray-code image $I_{gray_{t}}\left(x,y\right)$ is then combined with the header information $I_{h}\left(w,y\right)$, to form $I_{gray_{rgb}}\left(m,n\right)$, as shown in Equation (6), for transmission through the HFR projector as binary images:

(3) $$\;P=\sum_{i=0}^{n-1}b_{i}2^{i}=b_{0}2^{0}+b_{1}2^{1}+\ldots +b_{n-1}2^{n-1}\;,$$

(4) $$\;g_{i}=\left\{\begin{array}{cc} \;b_{i} & i=n-1\\ \;b_{i}⊕b_{i+1} & \;0\leq i\leq n-2 \end{array}\;,\right.$$

(5) $$\left[\begin{array}{c} I_{gray_{r}}\left(x,y\right)\\ I_{gray_{g}}\left(x,y\right)\\ I_{gray_{b}}\left(x,y\right) \end{array}\right]=I_{gray_{t}}\left(x,y\right)\;,$$

(6) $$I_{h}\left(w,y\right)+I_{gray_{t}}\left(x,y\right)=I_{gray_{rgb}}\left(x,y\right)\;,$$

## 5. Receiver Decoding System

**Figure 6:**
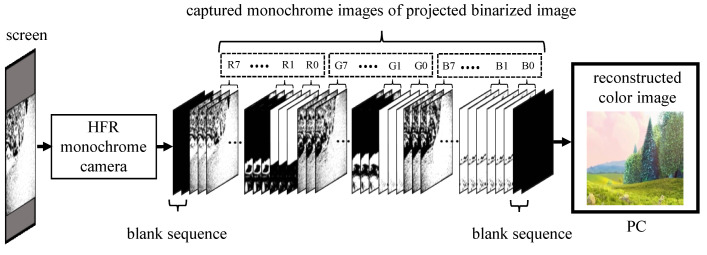
Receiver.

The receiver uses a monochrome HFR camera to decode the transmitted binary images of a 24-bit RGB image; its mechanism is shown in Figure 6. The transmitter and receiver systems are two separate systems, and they do not implement any hardware-based synchronization; therefore, software-based synchronization is used to synchronize them. After achieving synchronization, background subtraction is used to eliminate the ambient light on the projector screen in an indoor office room to extract the projected light intensity. The camera-projector alignment is corrected using camera calibration in post-processing to correct the orientation of the reconstructed image.

### 5.1. Software-Based Synchronization

**Figure 7:**
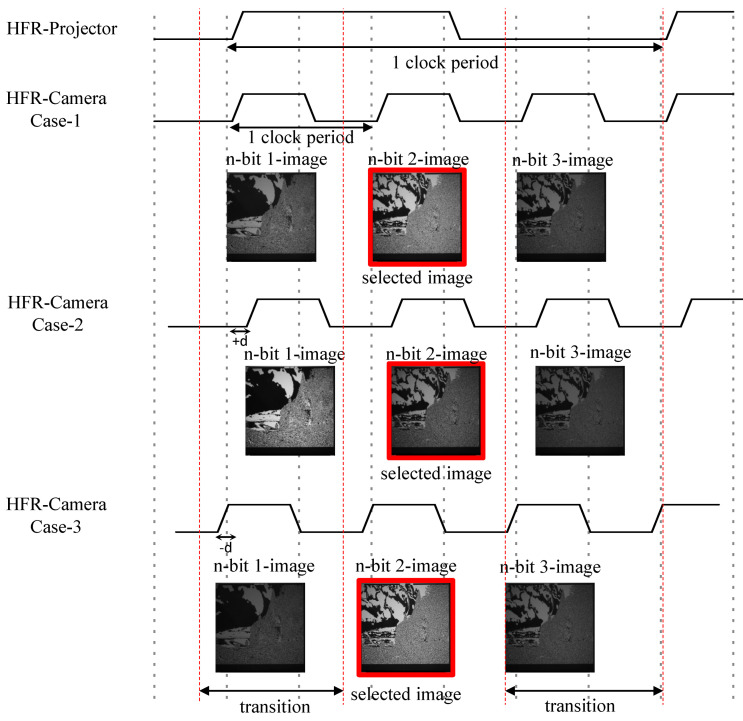
Image selection for software-based synchronization.

The HFR projector and HFR camera need to be synchronized to decode and reconstruct image sequences by capturing the binary images without any loss in pixel information. The HFR projector and HFR camera operate on their respective internal system clocks and are not connected to any common hard-wired external trigger; therefore, software-based synchronization is achieved at the receiver using the HFR camera. Using the Nyquist sampling theorem, which states that a continuous-time signal can be sampled and perfectly reconstructed from its samples if the waveform is sampled over twice as fast as its highest frequency component, software-based synchronization is achieved by setting the frame rate of the HFR camera to three times that of the HFR projector. Figure 7 describes the software-based synchronization method where three images are captured for a projected binary image and a total of three cases are observed. In case-1, the HFR camera starts capturing images at the same moment as the HFR projector projects images; thus, we can observe the satisfactory brightness of the first two images. In case-2, the HFR camera starts capturing images with a delay. Consequently, we obtain good brightness in the first two images. However, in case-3, the HFR camera starts capturing images before the HFR projector starts projecting; therefore, we can observe the satisfactory brightness of the second and third images. Thus, we selected the second image to reconstruct the original image because it has significant brightness compared to the other two images that are produced during the transitional stage.

### 5.2. Background Subtraction

The ambient light effect on the projector screen is eliminated by the background subtraction method with thresholding. In this method, a reference image is subtracted from the input image where the reference image is estimated using the global thresholding method by projecting the maximum and minimum intensities through the HFR projector onto the screen. Let $C_{in}\left(u,v\right)$ be the input image captured by the HFR camera, $C_{thr}\left(u,v\right)$ be a reference or threshold image, and $C_{bin}\left(u,v\right)$ be the binary image obtained after background subtraction. $C_{bin}\left(u,v\right)$ is calculated using Equation (7), where L(m,n) is the pixel value at (m,n) of $C_{in}\left(u,v\right)$ and thr(m,n) is the threshold value at (m,n) of $C_{thr}\left(u,v\right)$:

(7) $$\;C_{bin}\left(m,n\right)=\left\{\begin{array}{cc} 1 & \text{for}\;\;L(m,n)\geq thr(m,n)\\ 0 & \;\text{for}\;\;L(m,n)<thr(m,n) \end{array}\;,\right.$$

The threshold value thr(m,n) at (m,n) is calculated using Equation (8), where B(m,n) is the pixel value at (m,n) of $C_{in}\left(u,v\right)$, captured after projecting its maximum brightness, and D(m,n) is the pixel value at (m,n) of $C_{in}\left(u,v\right)$, captured after projecting a black image:

(8) $$\;thr(m,n)=\frac{B(m,n)}{2}+D(m,n)\;,$$

**Figure 8:**
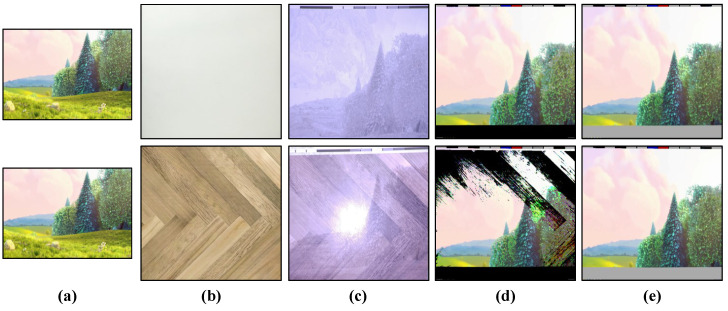
(**a**) original image; (**b**) background pattern; (**c**) projection on a background pattern; (**d**) reconstructed image without background subtraction; and (**e**) reconstructed image with background subtraction.

To evaluate the effectiveness of background subtraction, we used plain and patterned backgrounds as the projection screens. Initially, we projected the maximum and minimum brightness onto the projection screen to evaluate the background scene, which was subtracted from the input image. Figure 8a shows the input image used for projection, Figure 8b is the background used, Figure 8c shows the binarized image projected onto the background surface, Figure 8d illustrates the reconstructed image without background subtraction, and Figure 8e shows the reconstructed image with background subtraction. When using a plain white background, the global threshold value does not affect the entire reconstructed image because there is uniform reflectance of light throughout the surface. However, when a colored patterned background is used the threshold limit for each pixel varies owing to the reflectance of light, depending on the color it falls on. Therefore, we cannot use a global thresholding system. Figure 8d shows the reconstructed image, where a global thresholding technique is used; that is, a single threshold value is used for the entire image instead of a single pixel individually. The background subtraction method described above is used at the pixel level, where the threshold value is calculated for each pixel and, then, the image is reconstructed accordingly, as shown in Figure 8e.

### 5.3. Synthesizing 24-Bit RGB Image

The synthesizing or reconstruction of the original image is achieved by software-based synchronization of HFR projector–camera, background subtraction, and checking the header information. A threshold value T is required to extract data from the header information blocks which is constant and does not change dynamically as the threshold thr(m,n). The threshold value T determines the “0” and “1” bits of the header information and is calculated using Equation (9), where $B_{max}$ is the maximum brightness of a pixel in an image when projecting white light and $D_{min}$ defines the minimum brightness of a pixel in an image when projecting a black image:

(9) $$\;T=\frac{B_{max}+D_{min}}{2}\;,$$

To explain the process of synthesizing a 24-bit RGB color image, consider a gray-level input image $C_{in}\left(u,v\right)$ captured by the HFR camera and its corresponding binarized images of three channels as $C_{bin_{r(t)}}\left(u,v\right)$, $C_{bin_{g(t)}}\left(u,v\right)$ and $C_{bin_{b(t)}}\left(u,v\right)$ image which is then combined to form a single 24-bit RGB color image $C_{RGB}\left(u,v\right)$ as shown in Equation (10), where t is the bitplane number of 8-bit channels. The $C_{RGB}\left(u,v\right)$ image is an encoded gray-code-based image that is further decoded to a pure-binary-code-based image by using Equation (11) at the pixel level to obtain the reconstructed RGB color image $I_{RGB}\left(u,v\right)$:

(10) $$\left[\begin{array}{c} C_{bin_{r(t)}}\left(u,v\right)\\ C_{bin_{g(t)}}\left(u,v\right)\\ C_{bin_{b(t)}}\left(u,v\right) \end{array}\right]=C_{RGB}\left(u,v\right)\;0\leq t\leq 7\;,$$

(11) $$\;b_{i}=\left\{\begin{array}{cc} \;g_{i} & i=n-1\\ \;b_{i+1}⊕g_{i} & \;0\leq i\leq n-2 \end{array}\right.\;,$$

## 6. Image Quality in VLC

The image quality is a characteristic of an image that analyzes a set of measurable image quality attributes, such as image degradation and the amount of distortion or artifacts. Various physical properties, such as lens blur, display resolution, and refresh rate, affect the image quality, but are unlikely to change for a particular system. The perceived image quality in our system is compromised owing to the ambient light and the optics of the projector and camera systems. Image quality assessment is generally categorized into subjective and objective methods; for the proposed system, the objective method-based full reference metrics image quality assessment is used to evaluate the performance. For this image, registration is required between the reconstructed image and its reference image to evaluate the pixel-wise relationship between them. Therefore, image alignment or image registration is performed by warping the reconstructed images so that the features of the two images align perfectly. We used a plane projection surface for all experiments with different patterned backgrounds; only the geometric distortion was corrected and radiometric compensation was not considered. Quality measures, such as the peak-signal-to-noise ratio (PSNR), mean structural similarity index (MSSIM), and multi-scale structural similarity index (MS-SSIM) [79], were used to assess the image quality. PSNR was used to compare images with different dynamic ranges; it can be defined as the ratio of the maximum possible power of a signal and distortion. It has been expressed in Equation (12), where MSE is the mean-squared error and $MAX_{I}$ is the dynamic range of allowable pixel intensities:

(12) $$\;PSNR=10\cdot \log_{10}\left(\frac{MAX_{I}^{2}}{MSE}\right).$$

PSNR is easy to compute and has a good reduced reference model, but it does not match well with the human visual perceived quality. Here, the higher the PSNR value, the better the quality of the estimated image. Other methods based on the human visual system (HVS), such as SSIM and MS-SSIM, provide accurate results because they consider the human perception of image quality. The SSIM algorithm extracts the structural information from the field of view based on the HVS assumption. The pixels of the original image carry strong dependencies of the structure of a scene, which is independent of local luminance and contrast. Conversely, MSSIM is derived from SSIM by taking the mean of the SSIM index to evaluate the overall quality of the image:

(13) $$\;SSIM\left(x,y\right)=\frac{\left(2\mu_{x}\mu_{y}+C_{1}\right)\left(2\sigma_{xy}+C_{2}\right)}{\left(\mu_{x}^{2}+\mu_{y}^{2}+C_{1}\right)\left(\sigma_{x}^{2}+\sigma_{y}^{2}+C_{2}\right)},$$

(14) $$\;MSSIM\left(x,y\right)=\frac{1}{M}\sum_{j=1}^{M}SSIM\left(x_{j},y_{j}\right),$$

SSIM is a single-scale approach, but its performance depends mostly on appropriate viewing angles and the resolution of the display; it can be calculated using Equation (13), and Equation (14) represents the mean SSIM. This drawback of SSIM can be overcome by MS-SSIM, which is a novel synthesis-based approach to calibrate the parameters that weigh the relative importance between different scales; however, it is not very useful for badly blurred images. Equation (15) represents the MS-SSIM approach for image comparison at different scales. The measured error lies between 0 and 1, and the best quality value is 1. We used the PSNR and MS-SSIM methods to evaluate the image quality for our system:

(15) $$\;MS-SSIM\left(x,y\right)={\left[l_{M}\left(x,y\right)\right]}^{\alpha_{M}}\cdot \prod_{j=1}^{M}{\left[c_{j}\left(x,y\right)\right]}^{\beta_{j}}\cdot {\left[s_{j}\left(x,y\right)\right]}^{\gamma_{j}},$$

To evaluate the efficiency of the reconstructed images, a 5-bit frame number in the header information was used by assigning the frame number to each input frame, ranging from 1 to 32, thereby making a packet of 32 frames. These frame numbers were extracted at the receiver and checked for any loss within a packet of 32 images, which was calculated using Equation (16), where $F_{r}$ is the frame reconstruction efficiency and $S_{r}$ represents the successful frame reconstructed out of the total number of frames, $F_{t}$, within one packet of 32 frames. Thus, using the image quality assessment method, we can define the quality of images reconstructed at the receiver. In addition, the frame reconstruction efficiency explains the number of frames being reconstructed at the receiver and those being lost due to the bandwidth of the system and luminescence of the HFR-projector:

(16) $$\;F_{r}\;\left[\%\right]=\frac{S_{r}}{F_{t}}\times 100\;,$$

## 7. Experiments

**Figure 9:**
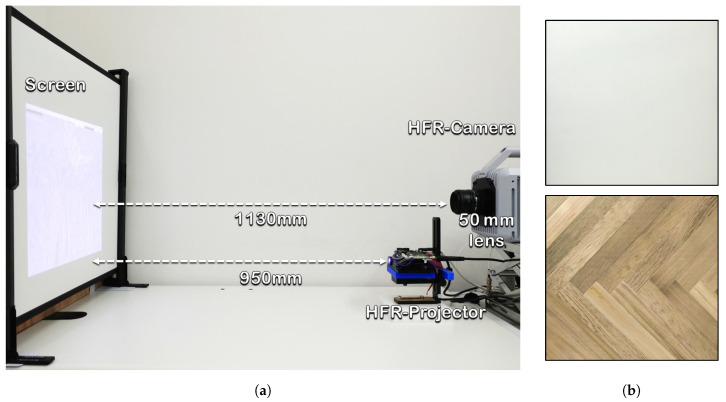
(**a**) overview of the HFR projector–camera system; (**b**) plain and patterned background.

The HFR-projector–camera system was set up in a controlled laboratory environment, and the corresponding experiments were conducted to evaluate the performance and image quality of the proposed VLC system. The projected video 590 × 1080 is a combination of 590 × 1060 gray-code images and 590 × 20 header information, projected in a bit plane sequence using the HFR projector. The bit plane sequence used for binary projection is shown in Figure 4b, where the green channel is projected first in a bit-plane sequence, followed by red and blue channel and the duration of exposure for each pattern is 960 μs. Therefore, the total duration for all bit-plane images is 23,040 μs, which is less than the vsync duration of the input video to avoid any frame loss. A 50-mm lens was mounted on the HFR camera, which was set to a maximum frame rate of 3125 fps. Therefore, the maximum frame rate of the HFR projector that can be used for projection for our system is 1041 fps, which is one-third of the HFR camera frame rate required for software-based synchronization. The experimental setup is shown in Figure 9a, where the distance between the HFR projector and screen is 950 mm and the projection display onto the screen is 448 mm × 415 mm. The distance between the HFR camera and screen is 1130 mm to ensure that the overall area of the projected video on the screen is captured by the camera. The experiments were performed on plain and patterned backgrounds, as shown in Figure 9b, for the proposed system for (a) a stored video sequence and (b) live video streaming from a USB camera. On the patterned background, the header information projected on a white background for the proper detection of header information. In addition, the indoor environment was illuminated with three different luminescence values (i.e., 0, 150, and 300 lux), using an external light source to evaluate the robustness of our system with respect to the ambient light.

### 7.1. Real-time Video Streaming—Stored Video Sequence

**Figure 10:**
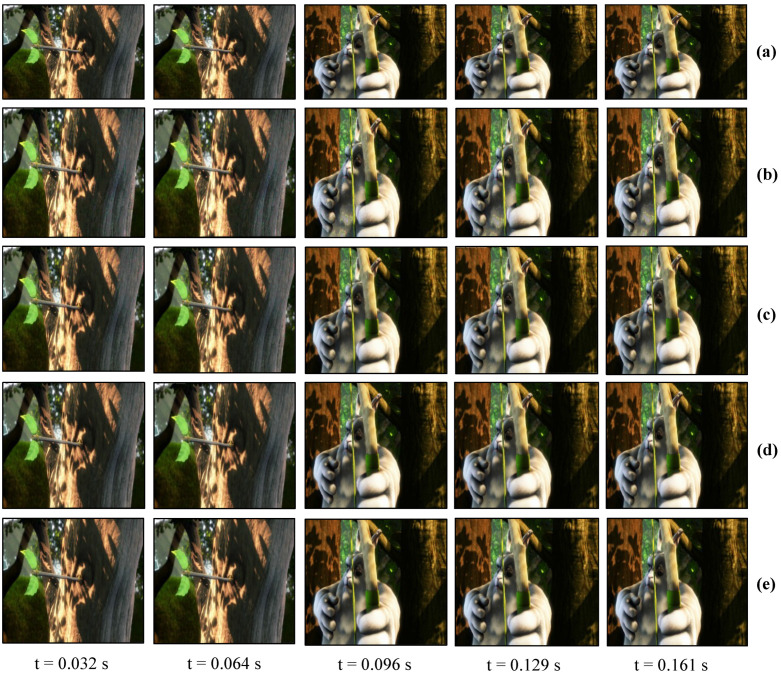
Reconstructed saved image sequence on plain background: (**a**) 1920 × 1080 input image; (**b**) 510 × 459 binary-code image without background subtraction; (**c**) 510 × 459 binary-code image with background subtraction; (**d**) 510 × 459 gray-code image without background subtraction; and (**e**) 510 × 459 gray-code image with background subtraction.

**Figure 11:**
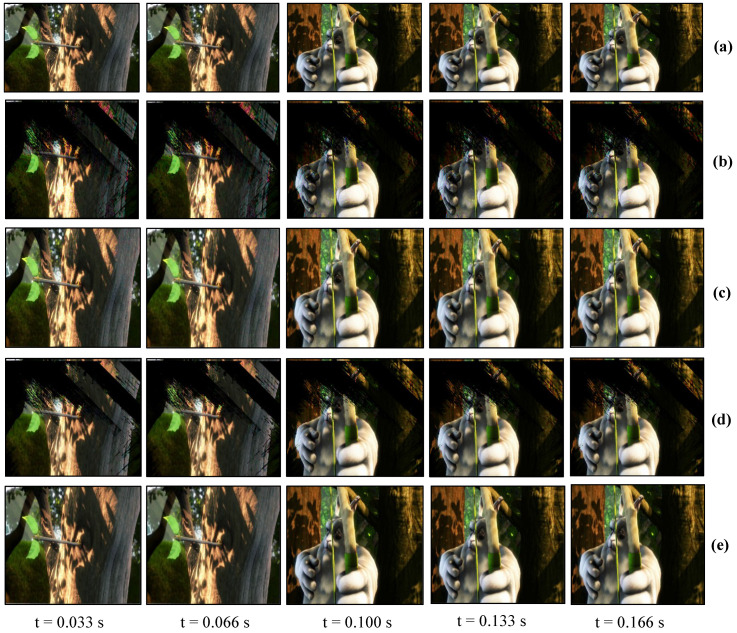
Reconstructed saved image sequence on a patterned background: (**a**) 1920 × 1080 input image; (**b**) 510 × 459 binary-code image without background subtraction; (**c**) 510 × 459 binary-code image with background subtraction; (**d**) 510 × 459 gray-code image without background subtraction; and (**e**) 510 × 459 gray-code image with background subtraction.

For a real-time video streaming experiment with a stored video sequence, we used the movie “BigBuckBunny” [80]. This experiment was performed to evaluate the performance and effectiveness of the binary and gray-code based encoding, and the background subtraction method. First, we estimated the background scene by projecting the maximum and minimum brightness for background subtraction. The pure-binary-code input images of 24-bit 1920 × 1080 RGB-color video were resized to 590 × 1060 which was then encoded to 590 × 1060 gray-code images, along with the addition of 590 × 20 header information, and projected using bit-plane or binary images at 1041 fps. The HFR camera captures 512 × 512 images and reconstructs the output image with a resolution of 510 × 459 by combining all bit-planes of a 24-bit RGB image sequentially. Figure 10 shows the comparison of the input image with binary-code-based projection and gray-code-based projection with background subtraction on a plain background. Figure 10a shows the full high definition input image 1920 × 1080 at 31 fps. Figure 10b,d depicts the reconstructed images 510 × 459 using pure-binary-code and gray-code respectively, without background subtraction. Figure 10c,e depicts the reconstructed images 510 × 459 using pure-binary-code and gray-code respectively, with background subtraction. Similarly, the experiments were performed for the patterned background, as shown in Figure 11. The images reconstructed with pure-binary-code exhibited artifacts due to the ambiguity of pixels with high spatial frequency; these artifacts were removed in the images reconstructed using gray-code-based transmission.

**Figure 12:**
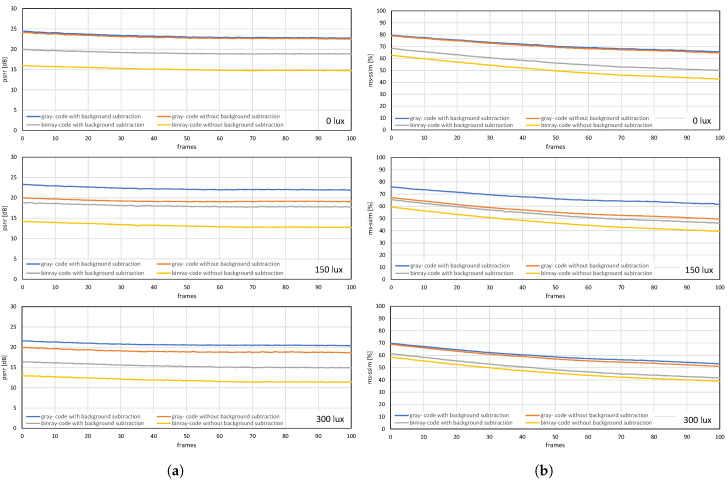
(**a**) PSNRs and (**b**) MS-SSIMs when a stored video sequence is streamed with pure-binary-code and gray-code images on a plain background.

**Figure 13:**
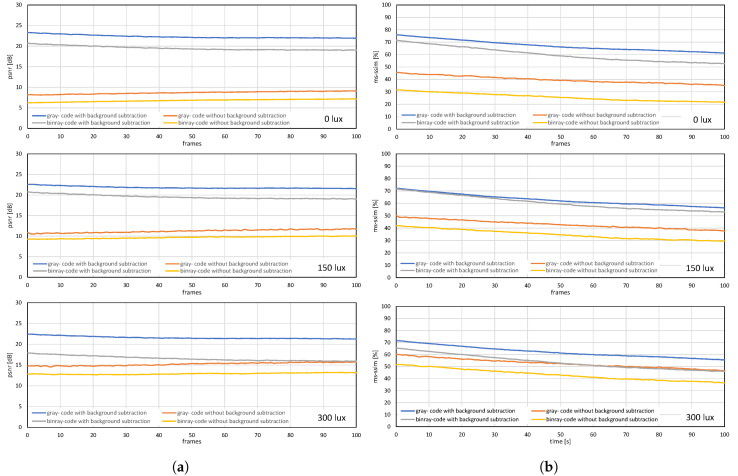
(**a**) PSNRs and (**b**) MS-SSIMs when a stored video sequence is streamed with pure-binary-code and gray-code images on a patterned background.

**Figure 14:**
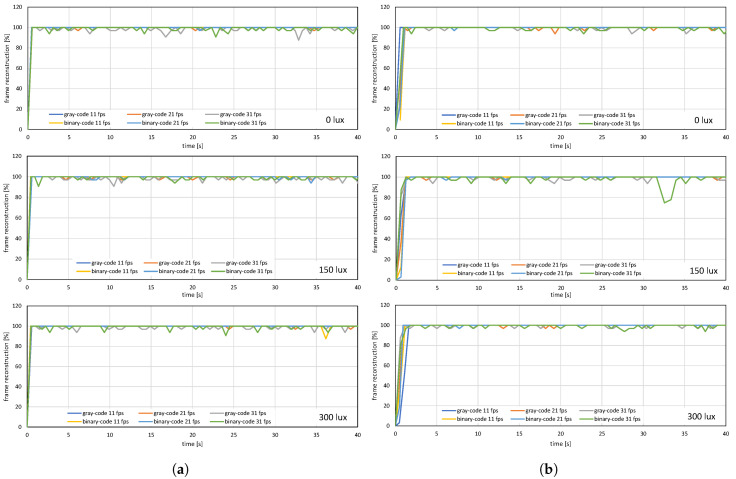
Frame reconstruction ratio when a stored movie is streaming on (**a**) plain background and (**b**) patterned background.

The image quality analysis and performance evaluation of the system measured under different on-screen luminescence of 0, 150, and 300 lux for three different input frame rates 11, 21, and 31 fps, for approximately a hundred consecutive frames are shown in Figure 12, Figure 13 and Figure 14.

**Figure 15:**
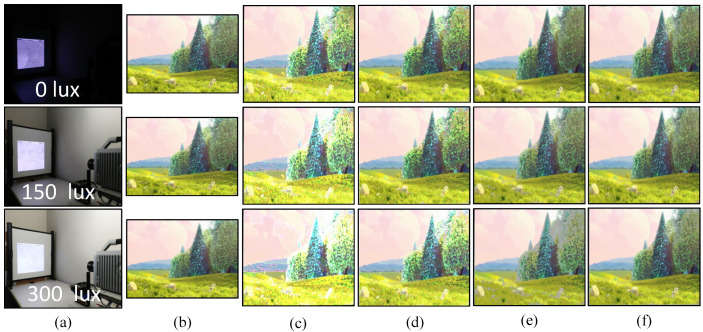
Plain background: (**a**) experiment scene at different illuminance levels; (**b**) 1920 × 1080 input images; (**c**) 510 × 459 images reconstructed using pure-binary-code without background subtraction; (**d**) 510 × 459 reconstructed images with pure-binary-code with background subtraction; (**e**) 510 × 459 reconstructed images using gray-code without background subtraction; and (**f**) 510 × 459 reconstructed images using gray-code with background subtraction.

**Figure 16:**
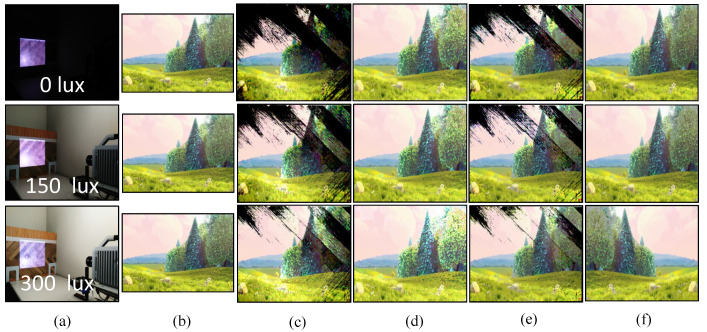
Pattern background: (**a**) experiment scene at different illuminance levels; (**b**) 1920 × 1080 input images; (**c**) 510 × 459 images reconstructed using pure-binary-code without background subtraction; (**d**) 510 × 459 reconstructed images with pure-binary-code with background subtraction; (**e**) 510 × 459 reconstructed images using gray-code without background subtraction; and (**f**) 510 × 459 reconstructed images using gray-code with background subtraction.

Figure 12 and Figure 13 shows the image quality by measuring the PSNRs and MS-SSIMs, where gray-code-based video reconstruction with background subtraction has a better quality index in comparison to others with respect to different luminescence values. We observed that the image quality was reduced on the patterned background compared to the plain background; however, the luminescence was increased to 300 lux, the patterned background with a slightly darker shade showed a better reconstructed image quality than the image reconstructed with gray-code on a plain background. Figure 14a,b show that, on plain and patterned backgrounds, the reconstructed image without background subtraction slightly differs from the image reconstructed with background subtraction, and there is a marginal difference between the images reconstructed using pure-binary-code and gray-code. However, as we increase the transmission frame rate, the reconstruction frame rate also starts dropping owing to the limited transmission bandwidth and due to the mixing of the channels of two consecutive frames at HFR generated by the HFR projector. Thus, we are discarding the images reconstructed using different frame numbers for an RGB channel sequence. Figure 15 and Figure 16 show the images reconstructed at different luminescence values on plain and patterned backgrounds, respectively. It is evident that the background subtraction method is significantly effective even when the luminescence is increased.

### 7.2. Real-Time Video Streaming—USB Camera

**Figure 17:**
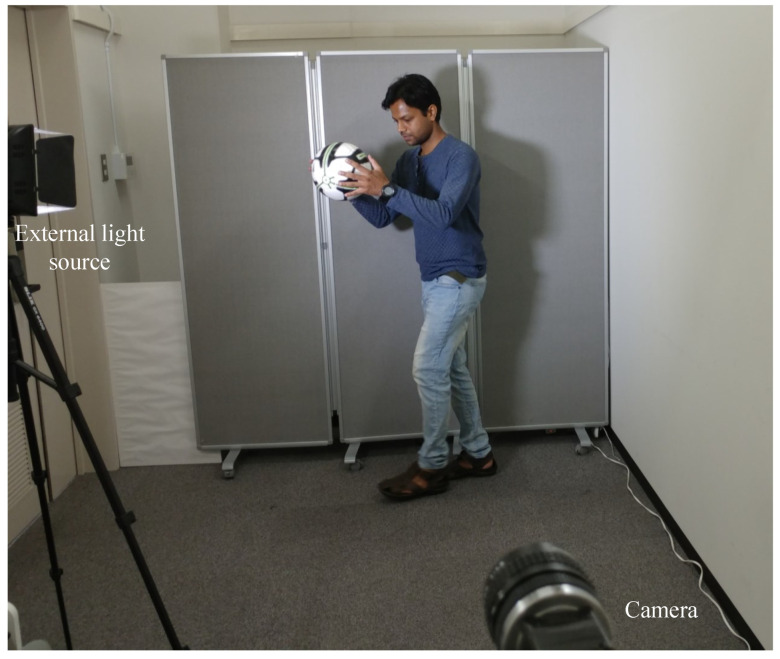
Experiment setup for HFR-projector–camera system using a USB camera as input.

**Figure 18:**
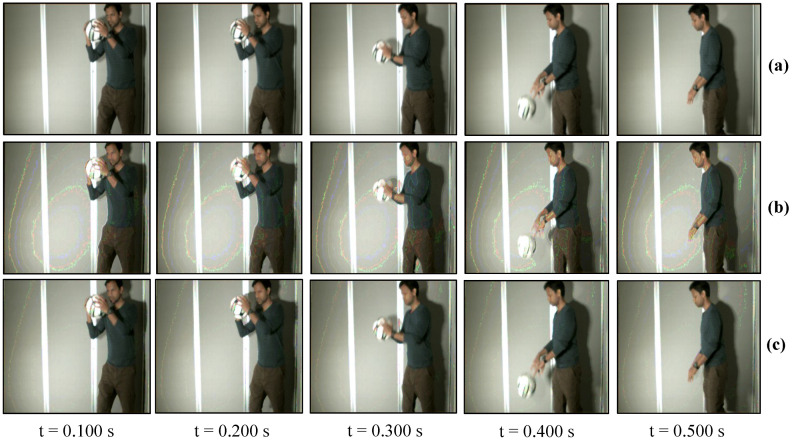
Reconstructed USB camera input image sequence on the plain background: (**a**) 640 × 480 input image; (**b**) 510 × 459 binary-code image without background subtraction; and (**c**) 510 × 459 binary-code image with background subtraction.

**Figure 19:**
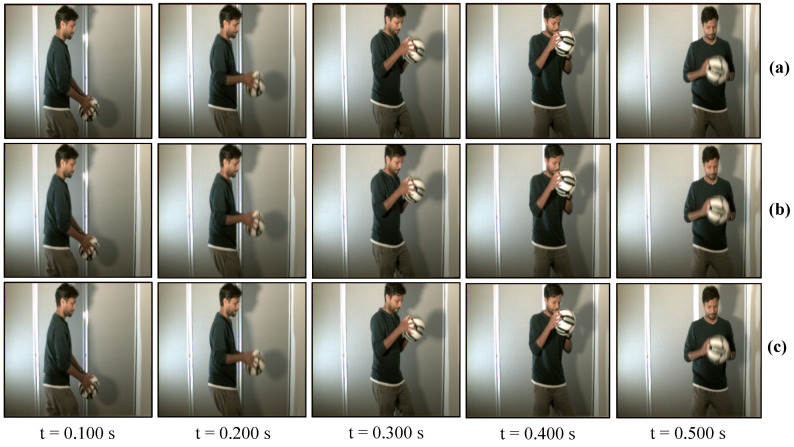
Reconstructed USB camera input image sequence on the plain background: (**a**) 640 × 480 input image; (**b**) 510 × 459 binary-code image without background subtraction; and (**c**) 510 × 459 binary-code image with background subtraction.

**Figure 20:**
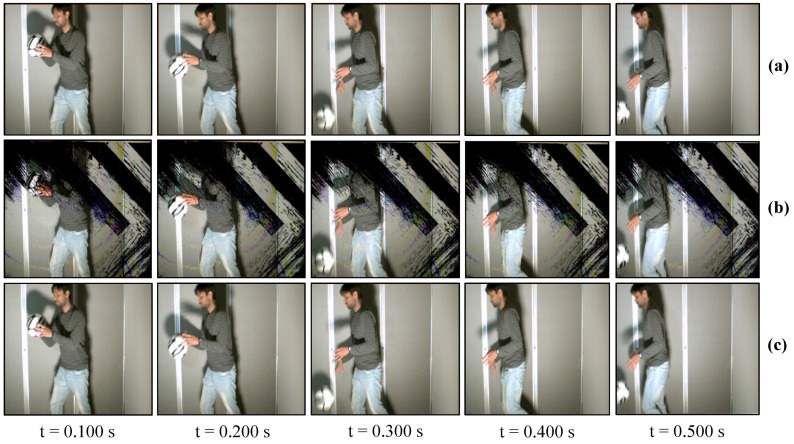
Reconstructed USB camera input image sequence on the pattern background: (**a**) 640 × 480 input image; (**b**) 510 × 459 binary-code image without background subtraction; and (**c**) 510 × 459 binary-code image with background subtraction.

**Figure 21:**
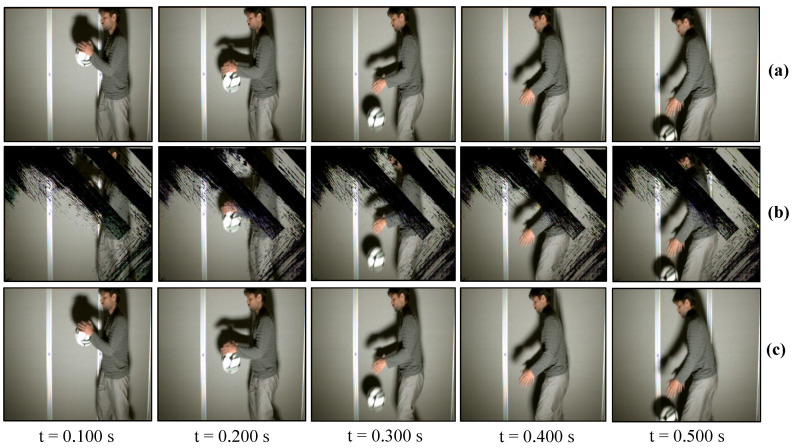
Reconstructed USB camera input image sequence on the pattern background: (**a**) 640 × 480 input image; (**b**) 510 × 459 binary-code image without background subtraction; and (**c**) 510 × 459 binary-code image with background subtraction.

**Figure 22:**
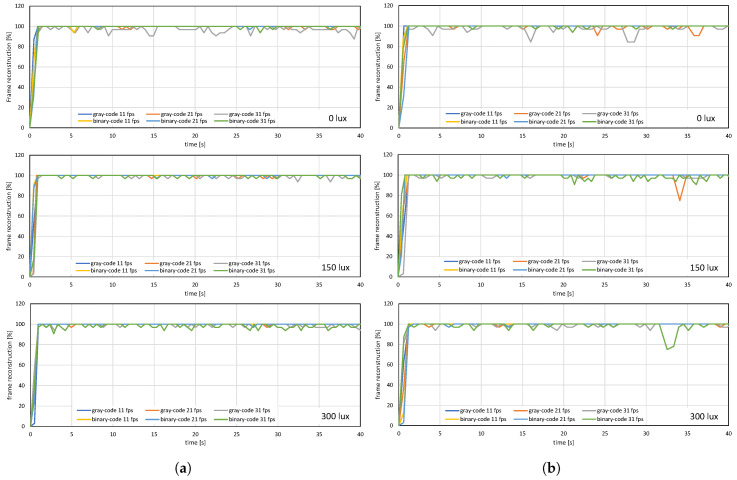
Frame reconstruction ratio when USB camera video is streaming on (**a**) plain background and (**b**) patterned background.

**Figure 23:**
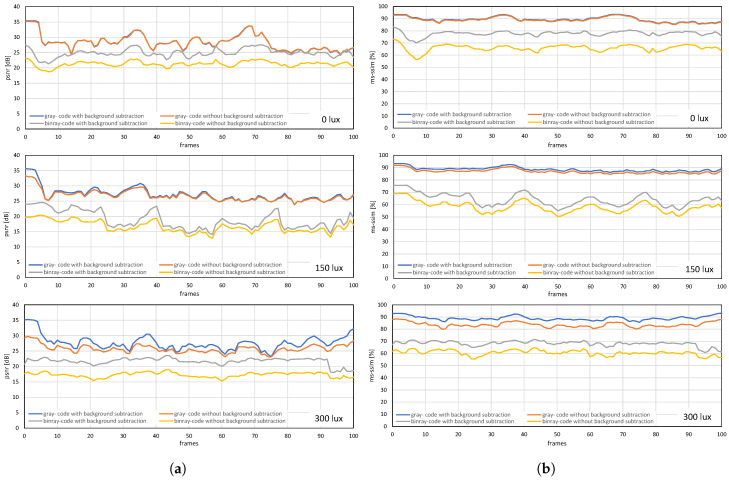
(**a**) PSNRs and (**b**) MS-SSIMs when USB camera video sequence is streamed with pure-binary-code and gray-code images on the plain background.

**Figure 24:**
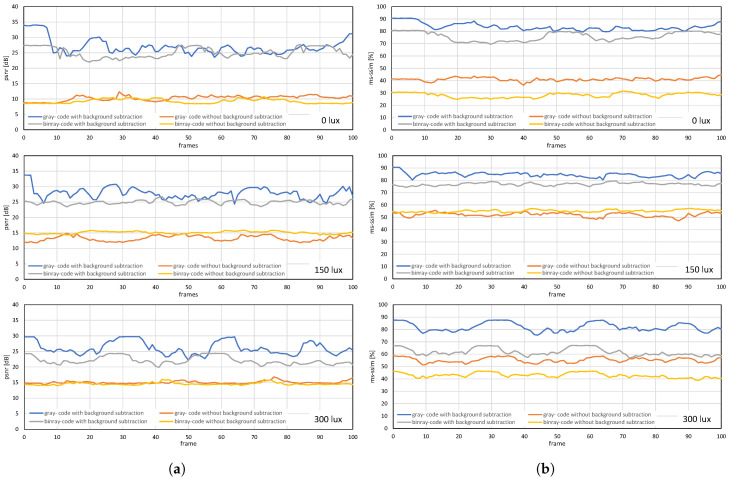
(**a**) PSNRs and (**b**) MS-SSIMs when USB camera video sequence is streamed with pure-binary-code and gray-code images on the patterned background.

The USB camera experiment was performed to verify the efficiency and performance of the real-time video streaming which can transmit the real-world information through the camera and verify its reconstruction at the receiver in real-time. In this experiment, the input video sequence was obtained from a USB camera (XIMEA, MQ003CG-CM), which is a 24-bit color camera, and its image resolution was set to 640 × 480 at 30 fps for transmission considering the conventional USB camera parameters. The experimental setup is shown in Figure 17. The experimental scene consists of a person throwing a football on the floor, and the HFR projector is set to 1041 fps with the same binary projection sequence as in Figure 4b, along with an HFR camera frame rate of 3125 fps. Figure 18 and Figure 19 show the comparison between pure-binary-code based and gray-code based reconstructed image sequences on the plain background, respectively. From Figure 18 and Figure 19, we can observe the reduction in artifacts when using gray-code-based encoding to reconstruct the image. Similarly, Figure 20 and Figure 21 show the comparison between pure-binary-code based and gray-code based reconstructed image sequences on the patterned background, respectively. The effectiveness of background subtraction method is evident from the reconstructed images in Figure 20 and Figure 21. Figure 22 shows the performance evaluation for the reconstructed USB camera video with three different input frame rates: 11, 21, and 31 fps for ambient luminescence of 0, 150, and 300 lux considering 100 consecutive images; their image qualities were measured using PSNR and MS-SSIM, as shown in Figure 23 and Figure 24 for the plain and patterned backgrounds, respectively. Figure 22 depicts that, with the increase in ambient light, a slight increase in frame loss can be observed at the receiver. Overall, the images reconstructed using gray-code with background subtraction have a higher image quality compared to other methods, and almost no frame loss is observed at 0 lux.

## 8. Conclusions

In this study, we developed a real-time video broadcasting system using VLC that can transmit saved and real-time USB camera videos through an HFR projector, operating at 1041 fps, and reconstruct the output color video using a monochrome HFR-camera at 3125 fps via software-based synchronization. In the proposed system, we evaluated the advantages of reconstructing the output images using gray-code over pure-binary-code based video transmission by removing the ambiguity occurring at gradients with pixels having a higher frequency component. Software-based synchronization is used to overcome the synchronization error between the HFR projector and HFR camera by considering the Nyquist sampling theorem. The use of thresholding-based background subtraction is efficient for eliminating the effect of ambient light and patterned background. Various experiments were conducted for real-time video broadcasting systems to evaluate the frame reconstruction at different fps and lux, wherein the frame loss was slightly increased with an increase in the frame rate and lux. However, the image quality of the reconstructed image was reduced as the luminescence of the ambient was increased, which was verified by comparing the image quality metrics, PSNR and MS-SSIM. The background subtraction method was found to be more effective for the patterned background than the plain background. Based on the experimental results, the system has limited bandwidth due to software-based synchronization, which can be increased in the future by perfectly synchronizing the HFR projector–camera system using an external trigger or visual feedback for the HFR camera.
